# Supplementary material for: A methodology for the design of experiments in computational intelligence with multiple regression models
Source: PeerJ. 2016 Dec 1;4:e2721. doi: 10.7717/peerj.2721 (PMC5136129; doi:10.7717/peerj.2721)
Supplement: Data S1 — Datailed results from UC Irvine Machine Learning Repository (Housing, Machine CPU, Wine Quality, Automobile and Parkinson) and the 3 Use Cases (Protein Corona, Gajewicz Metal Oxides and Aquatic Toxicity) [file peerj-04-2721-s001.zip › 6_UseCases/1_ProteinCorona/ENET.details.csv.repeatedcv.split8.pdf]

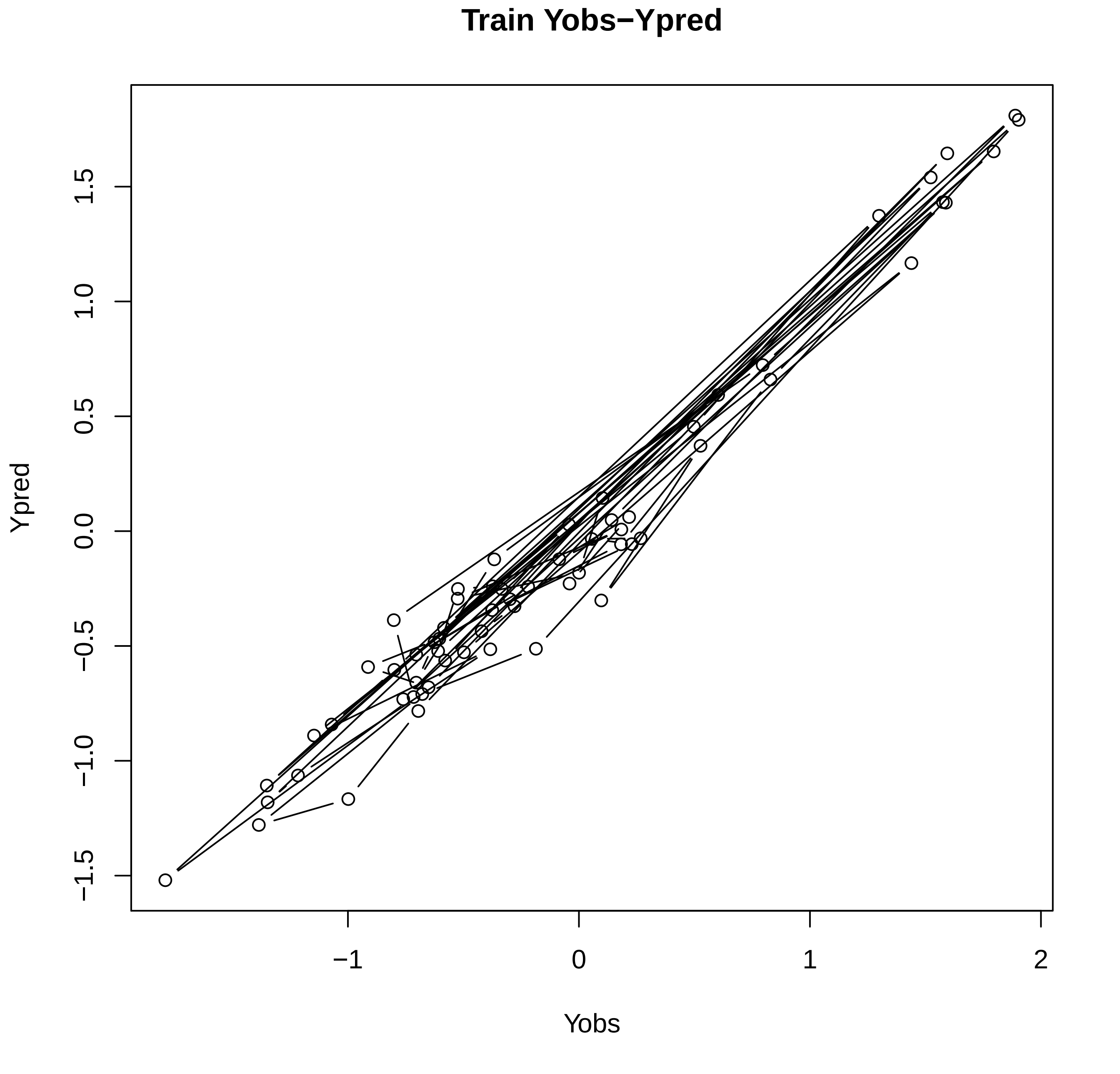
Train Yobs−Ypred
Ypred
Yobs
−1.5
−1.0
−0.5
0.0
0.5
1.0
1.5
−1
0
1
2

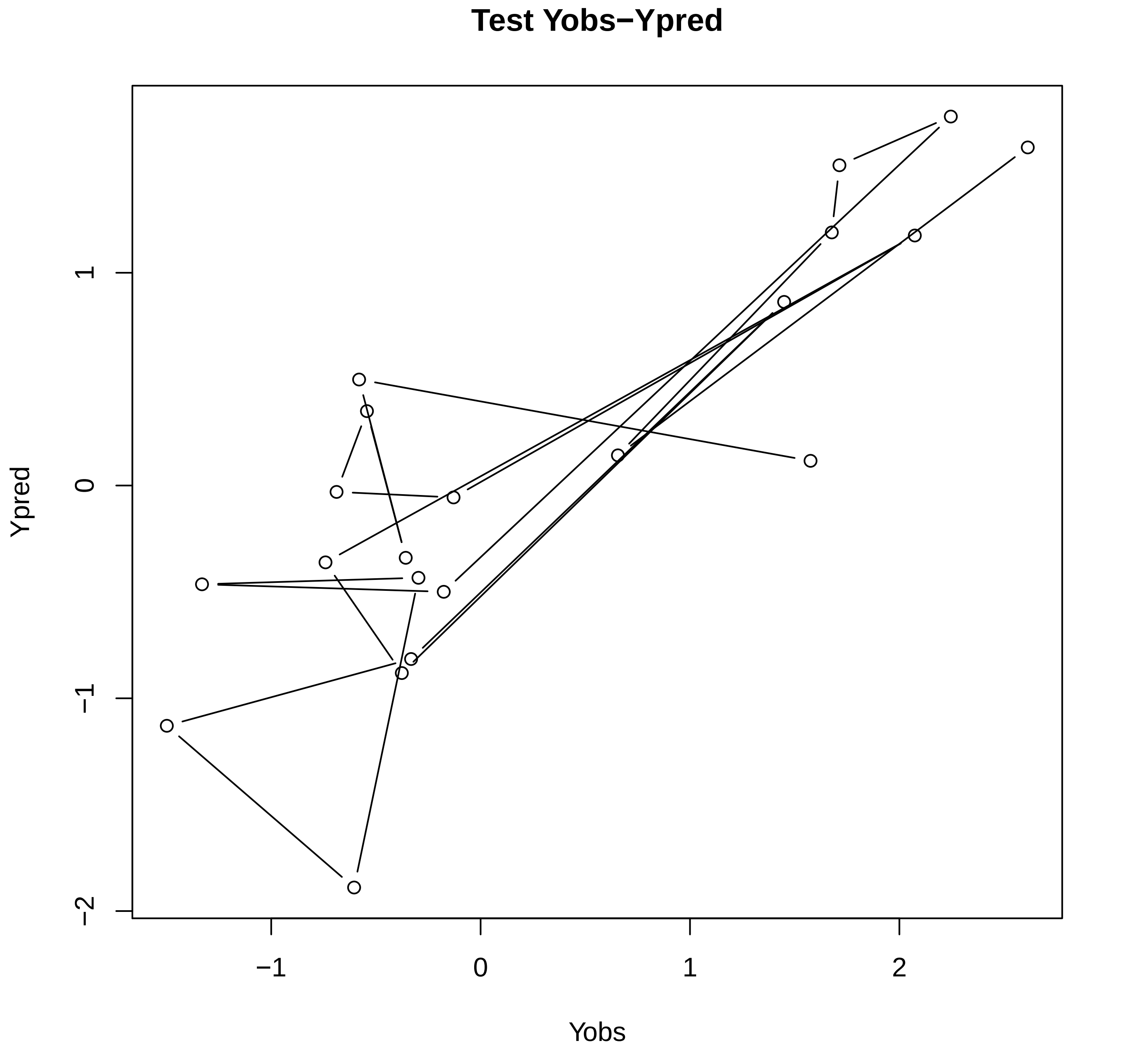
Test Yobs−Ypred
Ypred
Yobs
−2
−1
0
1
−1
0
1
2

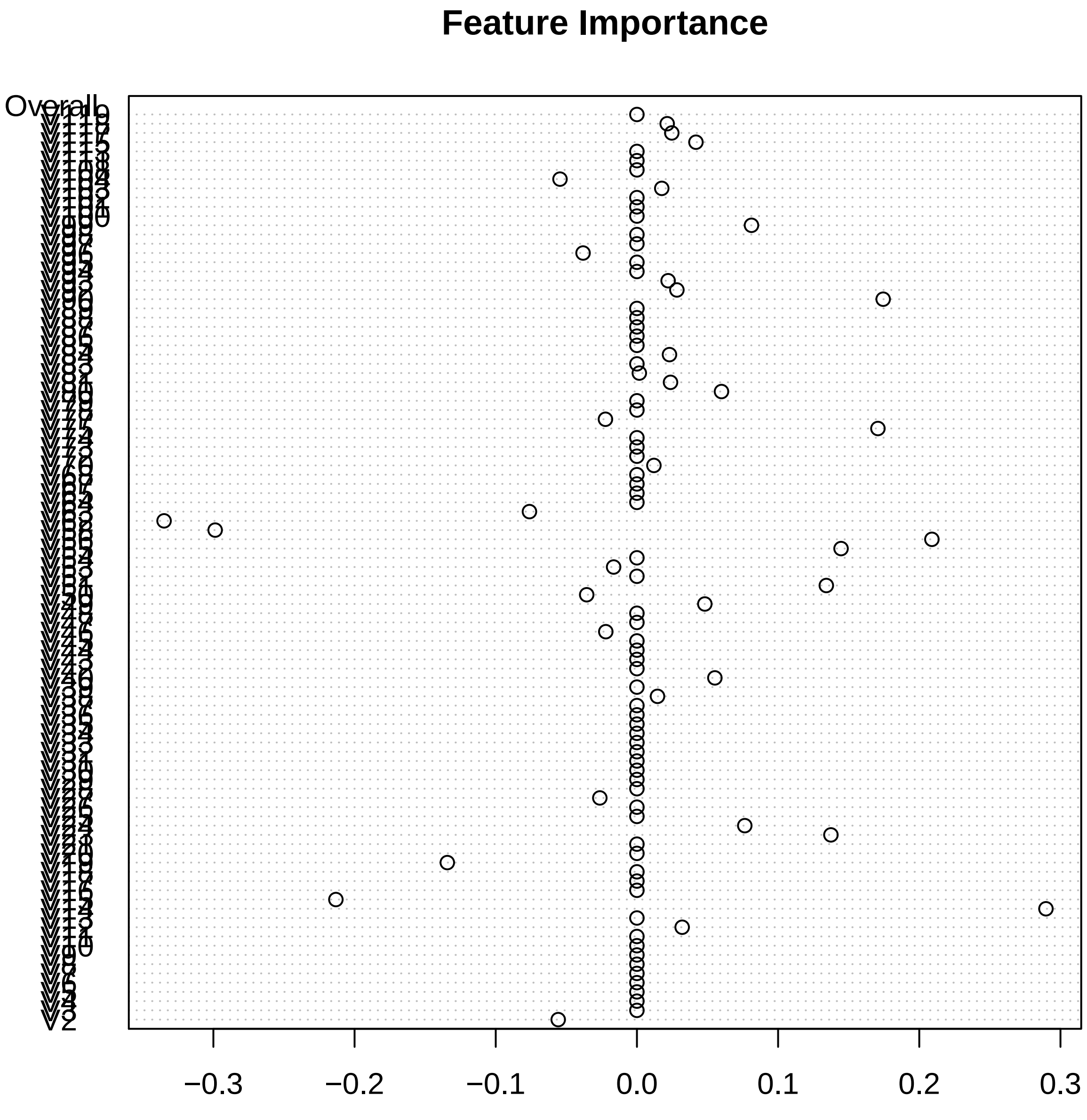
Feature Importance
Overall
V2
−0.3
−0.2
−0.1
0.0
0.1
0.2
0.3

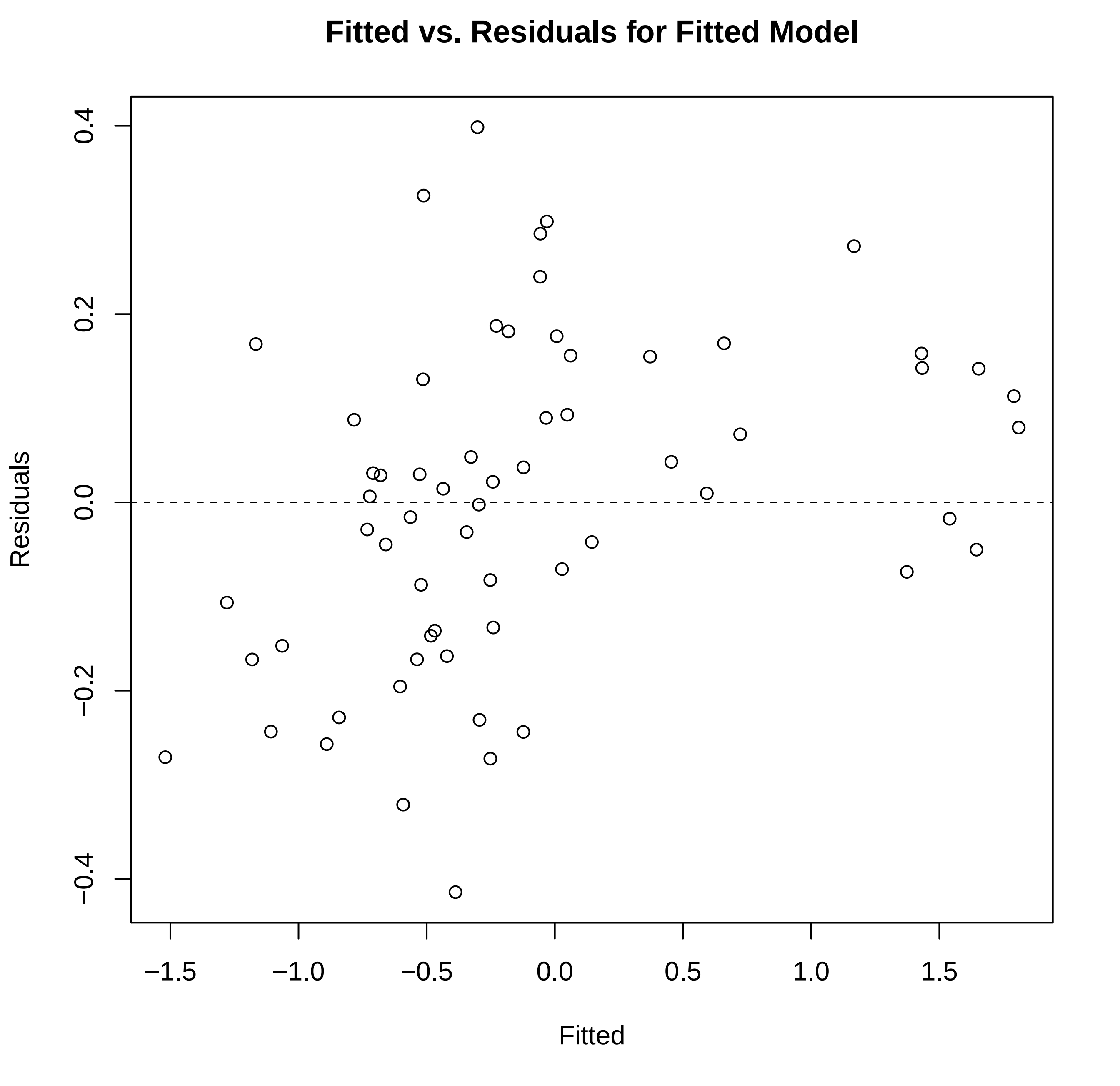
Fitted vs. Residuals for Fitted Model
Residuals
0.4
0.2
0.0
−0.2
−0.4
Fitted
−1.5
−1.0
−0.5
0.0
0.5
1.0
1.5
